# Supplementary material for: Inflammatory Mesenchymal Stem Cells Express Abundant Membrane-Bound and Soluble Forms of C-Type Lectin-like CD248
Source: Int J Mol Sci. 2023 May 31;24(11):9546. doi: 10.3390/ijms24119546 (PMC10253291; doi:10.3390/ijms24119546)
Supplement: Supplementary file 1 [file ijms-24-09546-s001.zip › ijms-2369032-supplementary.pdf]

International Journal of
Molecular Sciences

Supplemental Figure S1

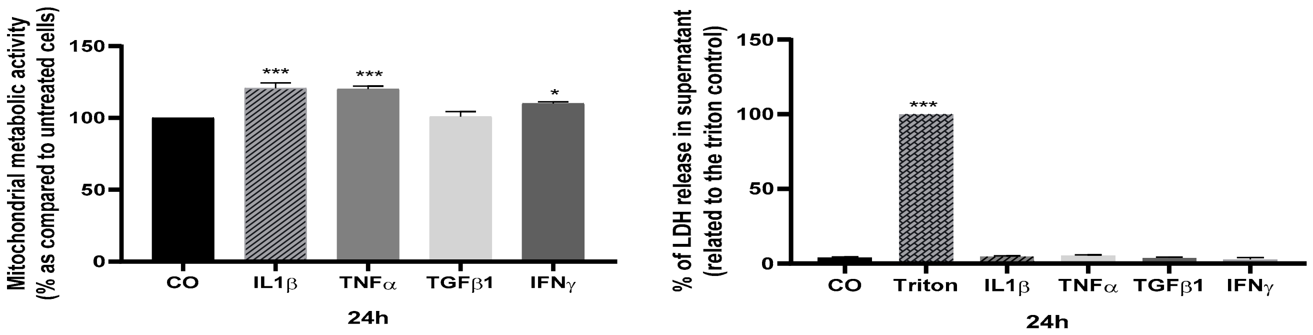

Figure S1. Synovial MSC were unstimulated (Co) or stimulated with recombinant cytokine/growth factors for 24h. MTT and LDH assays were performed (n=3) we found no cytotoxic activities in all tested conditions. * $p<0.05$, *** $p<0.001$.

Supplemental Figure S2

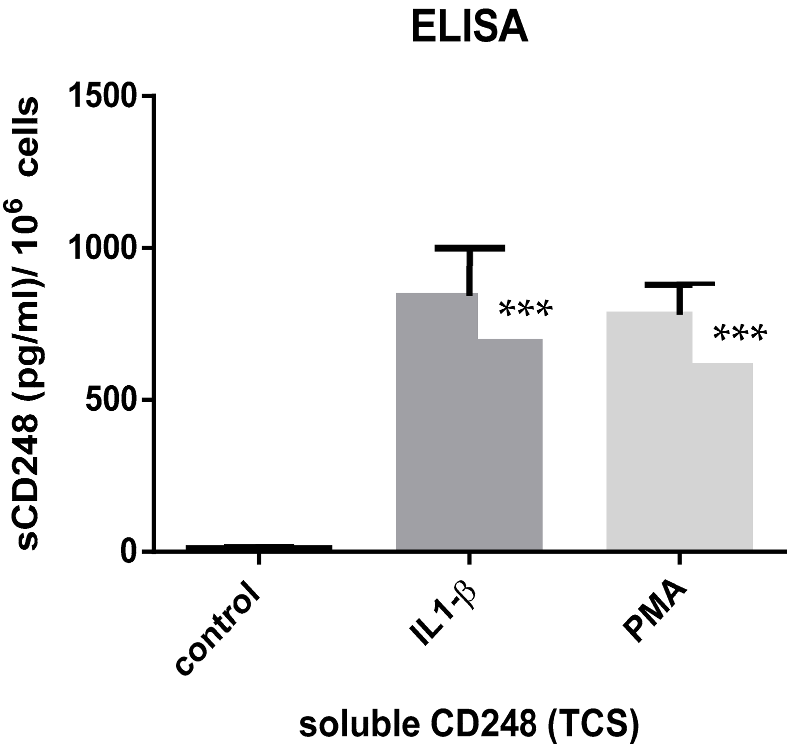

Figure S2. In house ELISA of TCS from synovial MSC (Sciencell), unstimulated (Control) or stimulated for 24h and tested (n=3) net (or diluted). Recombinant human CD248 Fc fusion protein was used as a standard. IL1β (20ng/ml, Peprotech) ; PMA (10ng/ml) phorbol ester, Sigma). *** $p<0.001$.
